# Supplementary material for: A methodology for the design of experiments in computational intelligence with multiple regression models
Source: PeerJ. 2016 Dec 1;4:e2721. doi: 10.7717/peerj.2721 (PMC5136129; doi:10.7717/peerj.2721)
Supplement: Data S1 — Datailed results from UC Irvine Machine Learning Repository (Housing, Machine CPU, Wine Quality, Automobile and Parkinson) and the 3 Use Cases (Protein Corona, Gajewicz Metal Oxides and Aquatic Toxicity) [file peerj-04-2721-s001.zip › 6_UseCases/2_GajewiczMetalOxides/RF.details.csv.repeatedcv.split2.pdf]

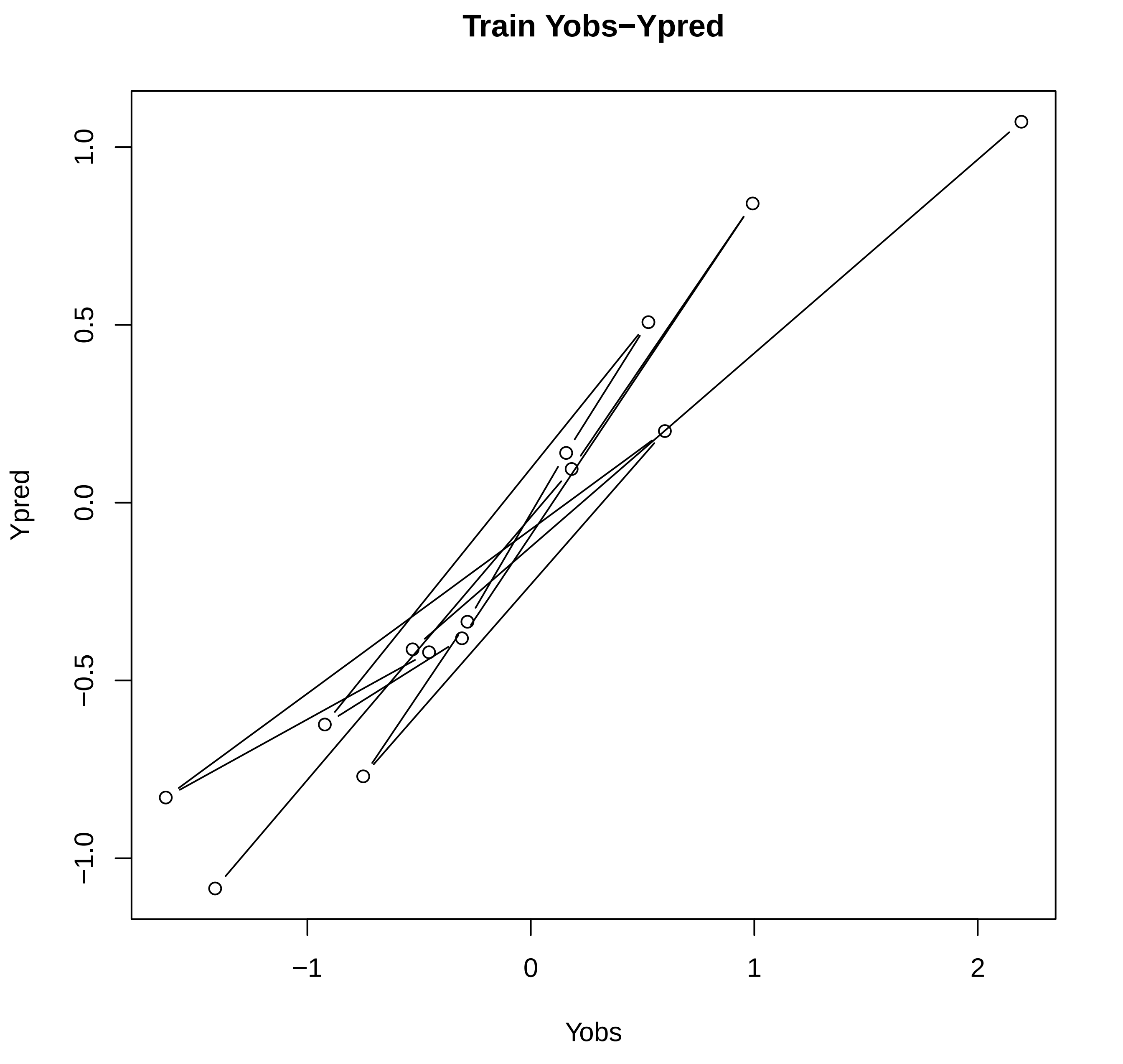
Train Yobs−Ypred
Ypred
Yobs
1.0
0.5
0.0
−0.5
−1.0
−1
0
1
2

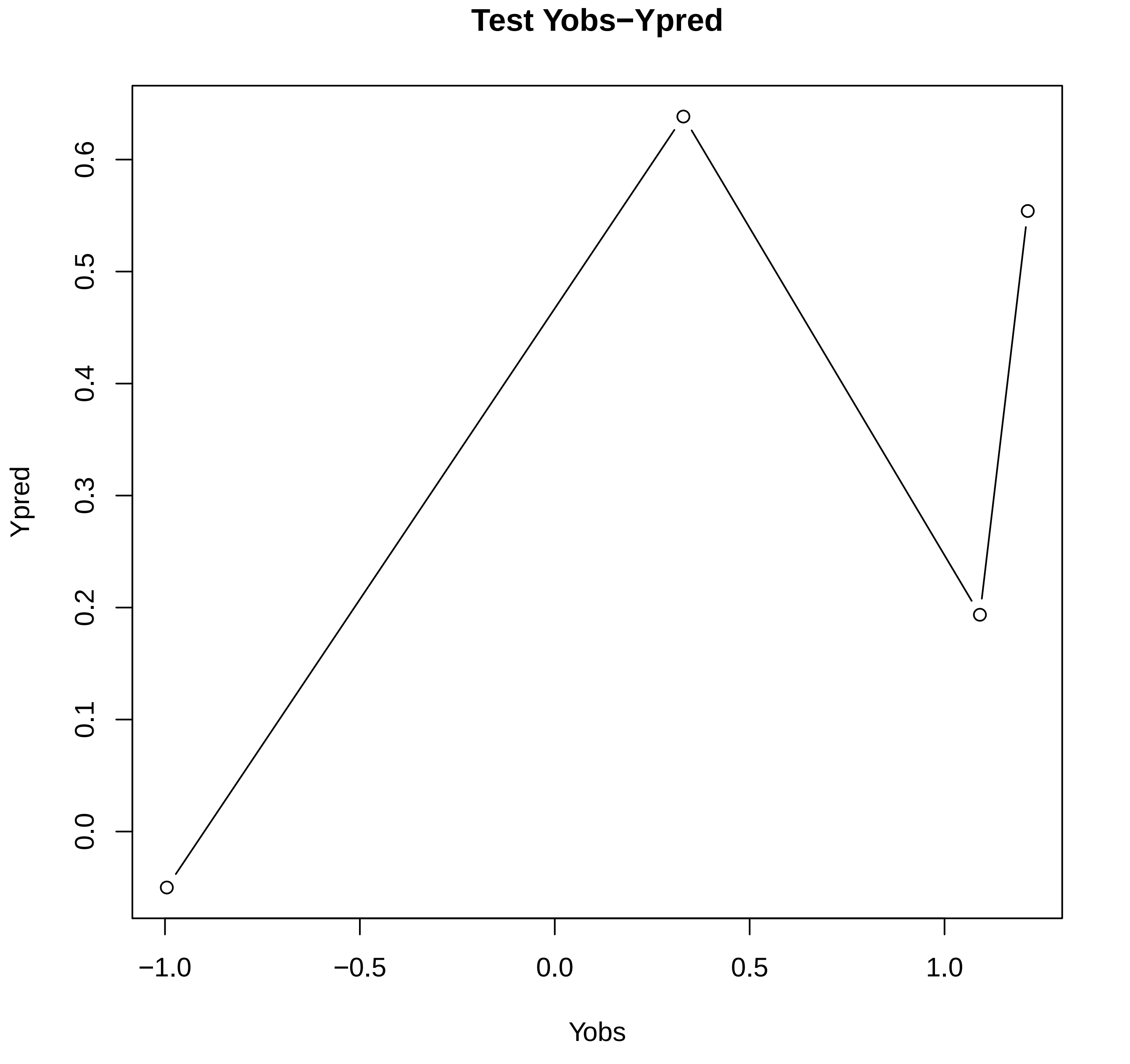
Test Yobs−Ypred
Ypred
0.0
0.1
0.2
0.3
0.4
0.5
0.6
−1.0
−0.5
0.0
0.5
1.0
Yobs

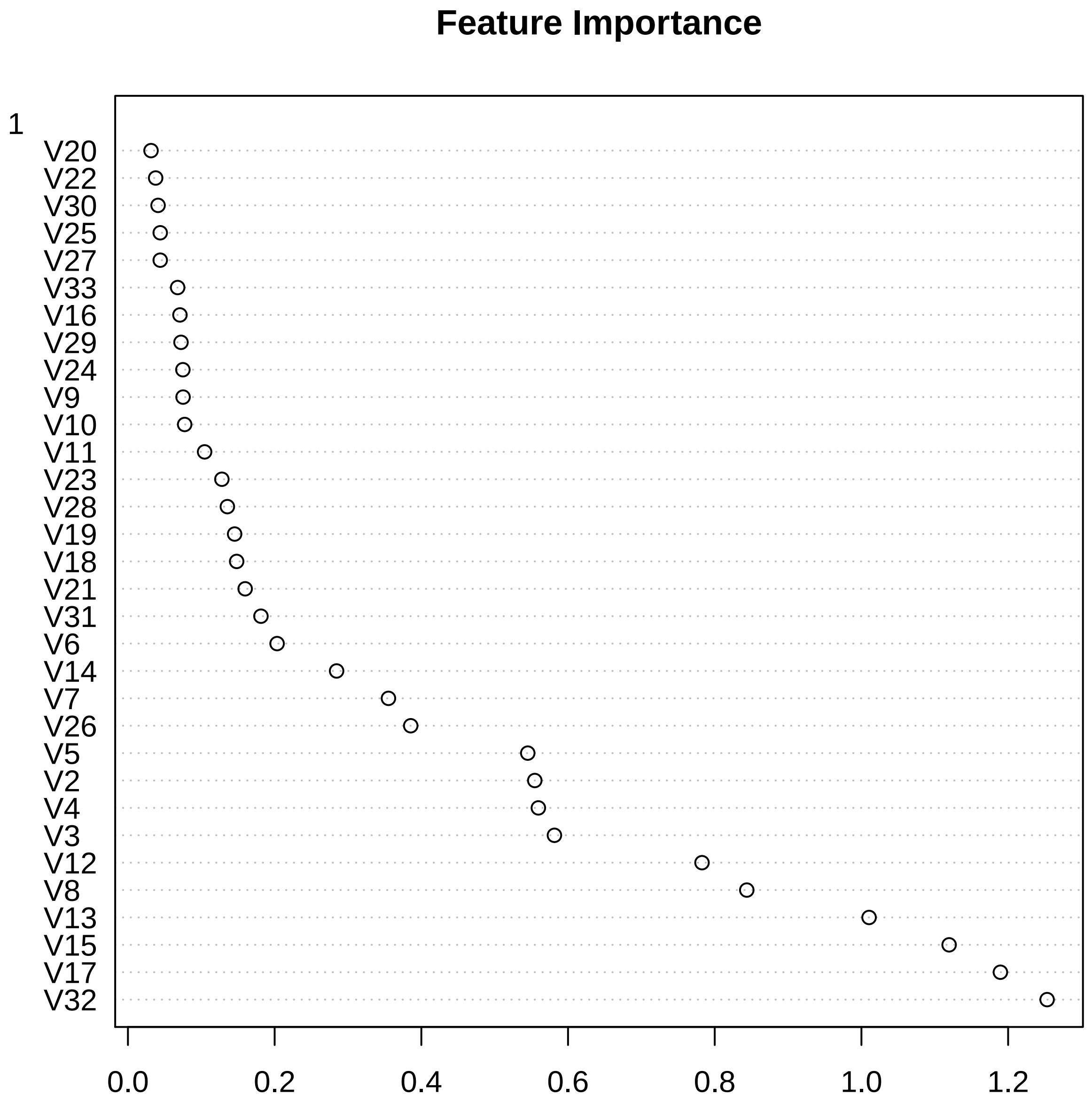
Feature Importance
1
V20
V22
V30
V25
V27
V33
V16
V29
V24
V9
V10
V11
V23
V28
V19
V18
V21
V31
V6
V14
V7
V26
V5
V2
V4
V3
V12
V8
V13
V15
V17
V32
0.0
0.2
0.4
0.6
0.8
1.0
1.2

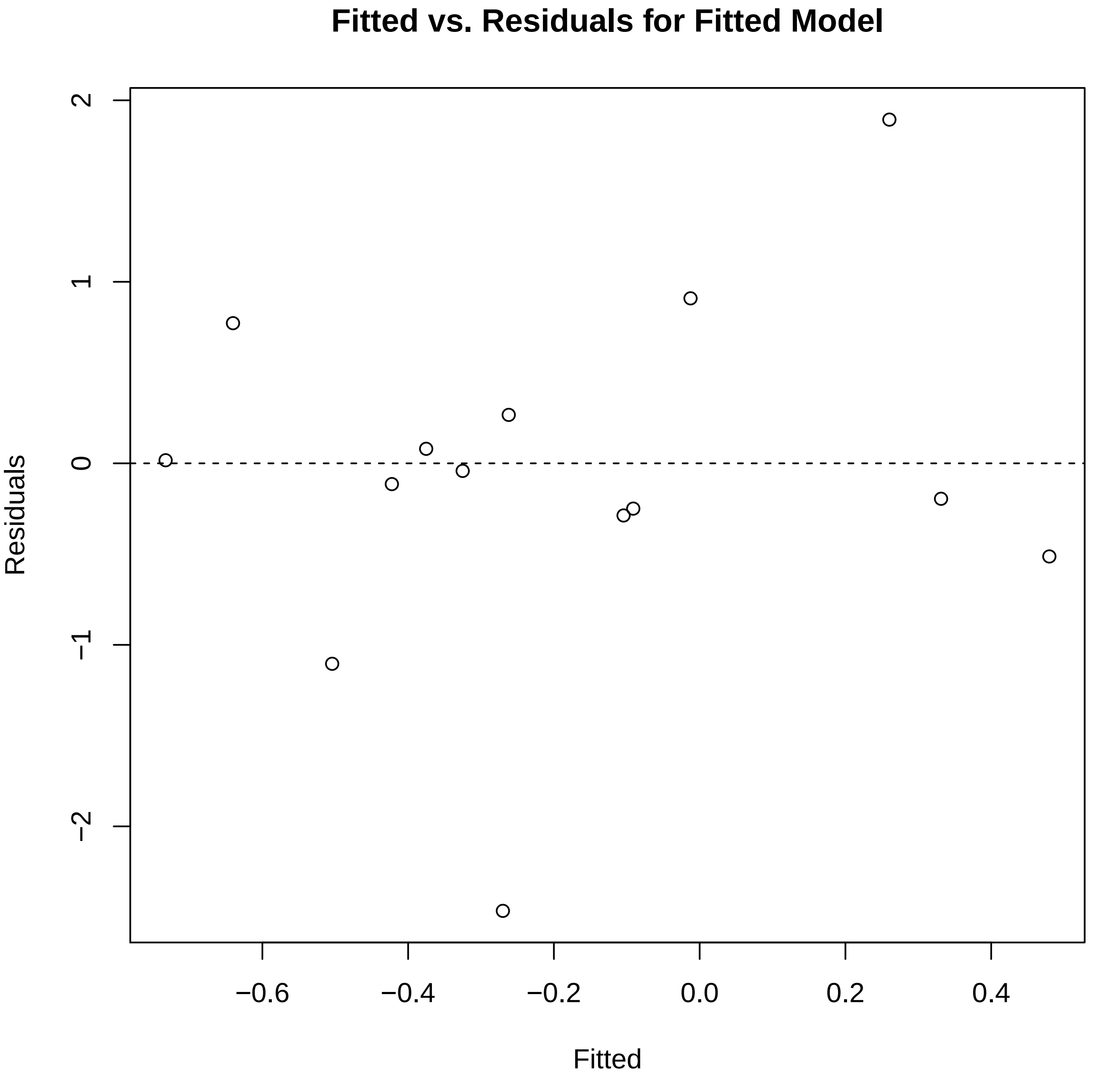
Fitted vs. Residuals for Fitted Model
Residuals
2
1
0
−1
−2
−0.6
−0.4
−0.2
0.0
0.2
0.4
Fitted
